# Recurrent Epigastric Pain and Non-bilious Vomiting Caused by Internal Herniation and Obstruction of the Jejunal Loop Constructed by Roux-en-Y Anastomosis: A Case Report of Postoperative Pregnancy With Congenital Biliary Dilatation

**DOI:** 10.7759/cureus.94599

**Published:** 2025-10-14

**Authors:** Yuya Tanaka, Satoru Ikenoue, Mototoshi Kato, Yuka Fukuma, Junko Tamai, Toshimitsu Otani, Yoshihumi Kasuga, Tomoshige Umeyama, Akihiro Fujino, Mamoru Tanaka

**Affiliations:** 1 Department of Obstetrics and Gynecology, Keio University School of Medicine, Tokyo, JPN; 2 Department of Pediatric Surgery, Keio University School of Medicine, Tokyo, JPN

**Keywords:** congenital biliary dilatation, intestinal obstruction, postoperative pregnancy, roux-en-y limb, roux-en-y reconstruction

## Abstract

Congenital biliary dilatation (CBD) requires Roux-en-Y hepaticojejunostomy in childhood. We experienced a rare case of postoperative pregnancy of CBD with typical symptoms of ileus but with normal brown stool, due to the internal hernia and obstruction of the Roux-en-Y limb.

After a Roux-en-Y bypass surgery at the age of two, a 35-year-old woman had unrelenting epigastric pain and non-bilious vomiting for one week at 35 weeks’ gestation. During the cesarean section, internal herniation of the Roux-en-Y limb caused by fibrous bands extending from the greater omentum was revealed, which was resected to relieve the intestinal obstruction. The patient was discharged from the hospital on the sixth postoperative day.

When pregnant women with a history of Roux-en-Y reconstruction present with epigastric pain and recurrent non-bilious vomiting but with normal stool, intestinal obstruction of the Roux-en-Y limb should be considered to prevent adverse perinatal outcomes.

## Introduction

Congenital biliary dilatation (CBD), also known as a choledochal cyst, is characterized by congenital dilatation of the bile ducts [1]. The most commonly performed surgery for CBD is resection of the extrahepatic dilated bile duct and Roux-en-Y hepaticojejunostomy [1,2]. During this procedure, the dilated extrahepatic bile duct is resected, the jejunum is separated, and the Roux-en-Y limb is anastomosed to the intrahepatic bile duct [1,2]. This approach prevents regurgitation of bile and pancreatic juice, as bile flows into the united intestinal duct and pancreatic juice flows normally through the papilla Vater into the duodenum [2].

In gastric and biliary surgery with Roux-en-Y reconstruction, the incidence of intestinal obstruction due to internal hernia has been reported as a late postoperative complication, with an incidence rate of 0.1% to 0.3% [3,4]. Especially during pregnancy, the risk of intestinal obstruction stemming from internal hernia is thought to be further increased because of heightened intra-abdominal pressure and cephalic bowel displacement caused by an enlarged uterus, increased sphincter of Oddi contraction due to progesterone, and relaxation of the gallbladder owing to estrogen and progesterone [5].

However, to our knowledge, there are no previous reports of pregnant women with Roux-en-Y hepatic jejunal anastomosis for CBD who developed intestinal obstruction due to internal hernia of the Roux-en-Y limb. In this report, we describe a case of postoperative CBD pregnancy with recurrent epigastric pain and nonbiliary vomiting, but with normal brown stool, caused by an internal hernia of the Roux-en-Y limb.

## Case presentation

**Figure 1:**
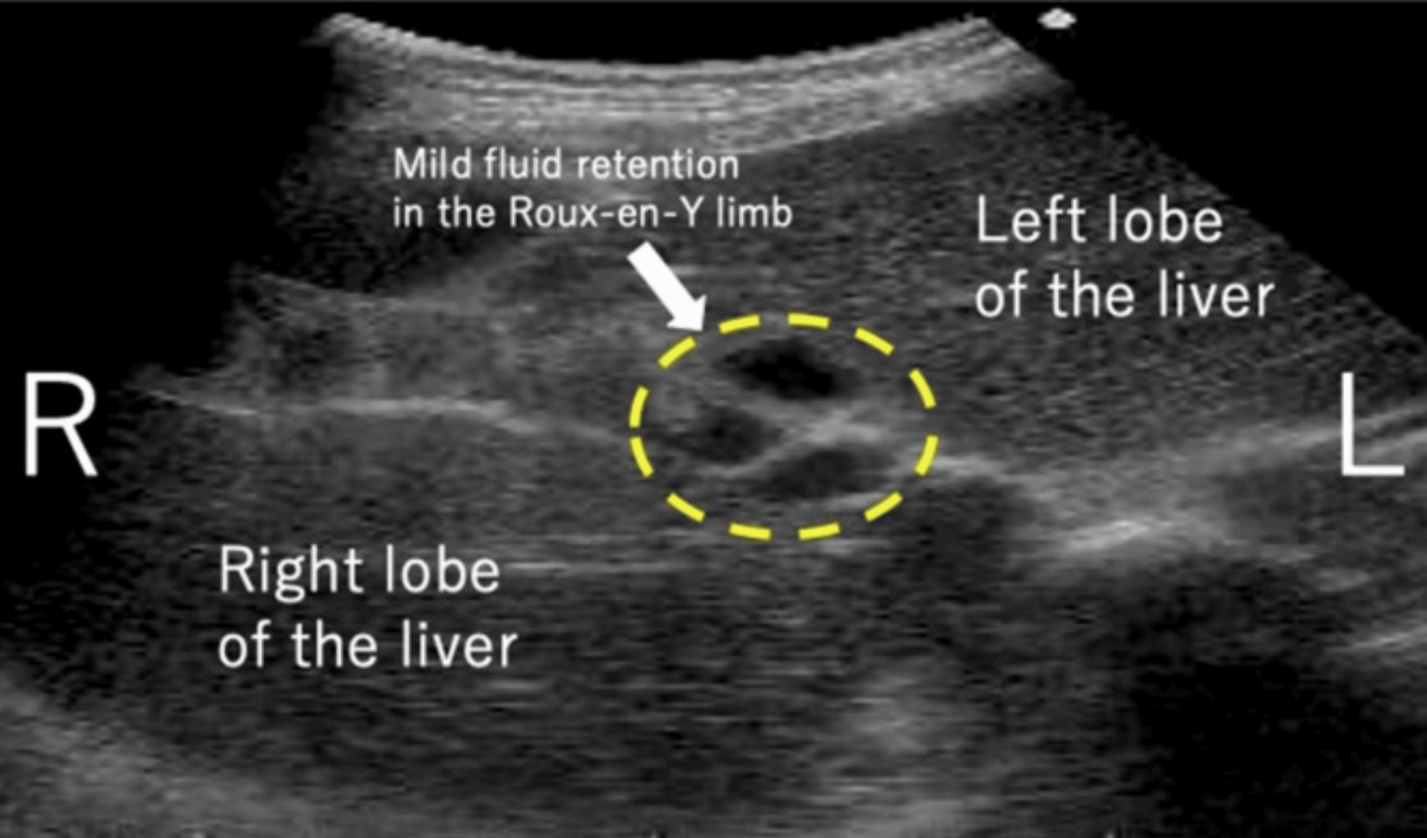
Abdominal ultrasonography. The white arrowhead represents mild fluid retention in the Roux-en-Y limb at the porta hepatis.

**Figure 2:**
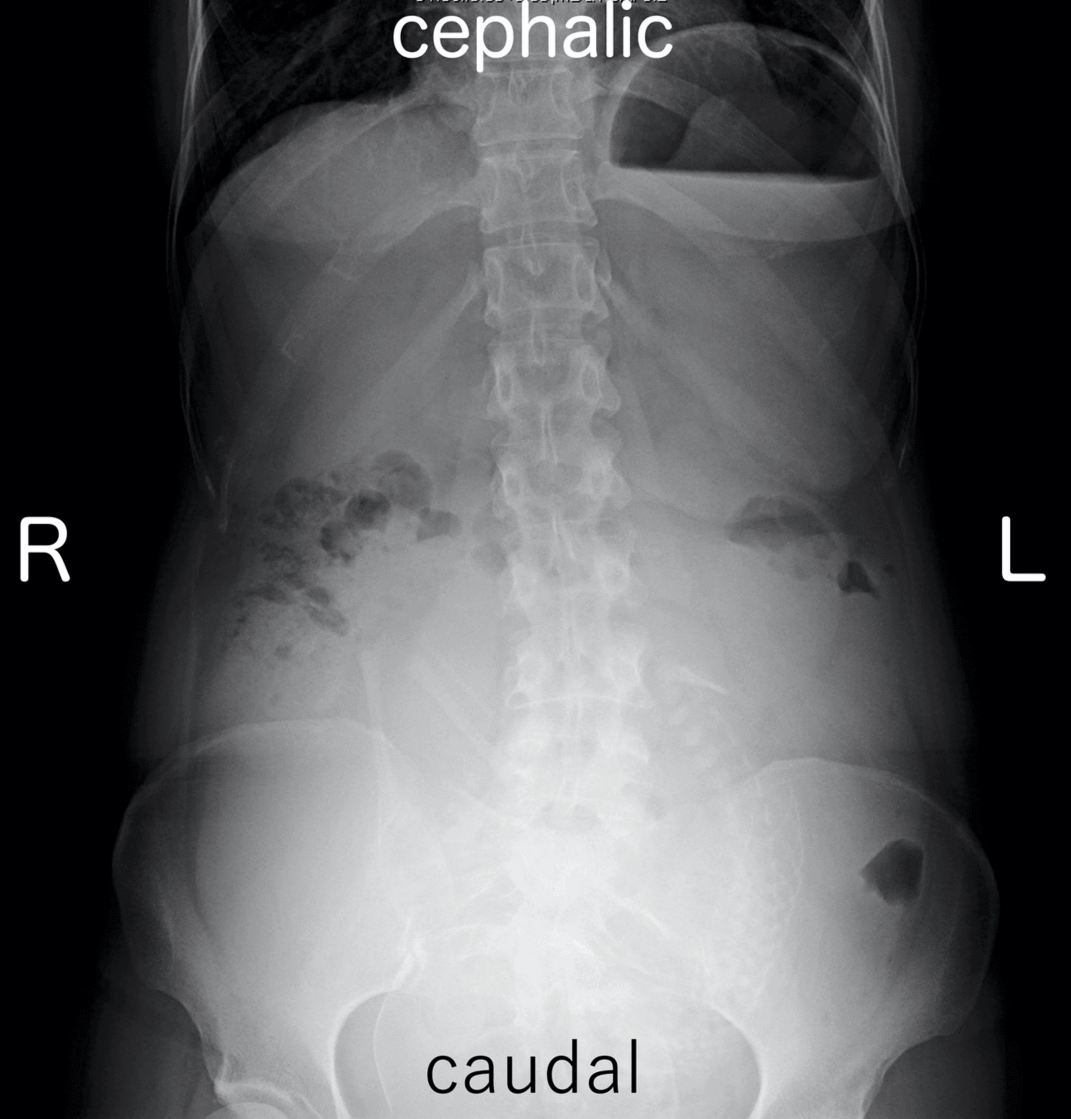
Abdominal X-ray in the upright position. There is no sign of niveau formation and distal small bowel gas.

A 35-year-old woman, G1P0, presented with a medical history of CBD. At the age of two, she underwent hepaticojejunostomy with Roux-en-Y reconstruction after resection of the dilated extrahepatic bile duct. She obtained a spontaneous pregnancy and received prenatal care in our hospital from the beginning of the pregnancy. At 35 weeks and one day of gestation, she visited our emergency department due to epigastric pain and recurrent non-bilious vomiting. Her bowel movement was regular and showed normal brown stool. Vital signs were as follows: body temperature 35.8°C, blood pressure 108/76 mmHg, and pulse rate 83 beats per minute. Physical examination revealed spontaneous pain and mild tenderness in the epigastric region, while the abdomen was soft without signs of muscular defense or rebound tenderness. Obstetric examination showed a closed cervix with a cervical length of 30 mm on vaginal ultrasonography. Fetal heart rate monitoring indicated a reassuring fetal status. Blood tests showed a white blood cell count of 11,700/μL, C-reactive protein of 0.08 mg/dL (indicating an absent inflammatory response), and normal liver function with aspartate aminotransferase (AST) 28 IU/L, alanine aminotransferase (ALT) 23 IU/L, and total bilirubin 1.5 mg/dL. Abdominal ultrasonography revealed mild fluid retention in the Roux-en-Y limb at the porta hepatis (Figure 1). An abdominal X-ray, performed to rule out severe intestinal obstruction, showed displacement of the entire intestine due to the pregnant uterus, with partial colonic gas but no obvious signs of niveau formation or distal small bowel gas (Figure 2). Based on these findings, gastroesophageal reflux caused by compression from an enlarged uterus was suspected, and the patient was admitted to our hospital. Because there were limited signs suggesting intestinal obstruction, computed tomography was not performed to avoid unnecessary radiation exposure to the fetus.

**Figure 3:**
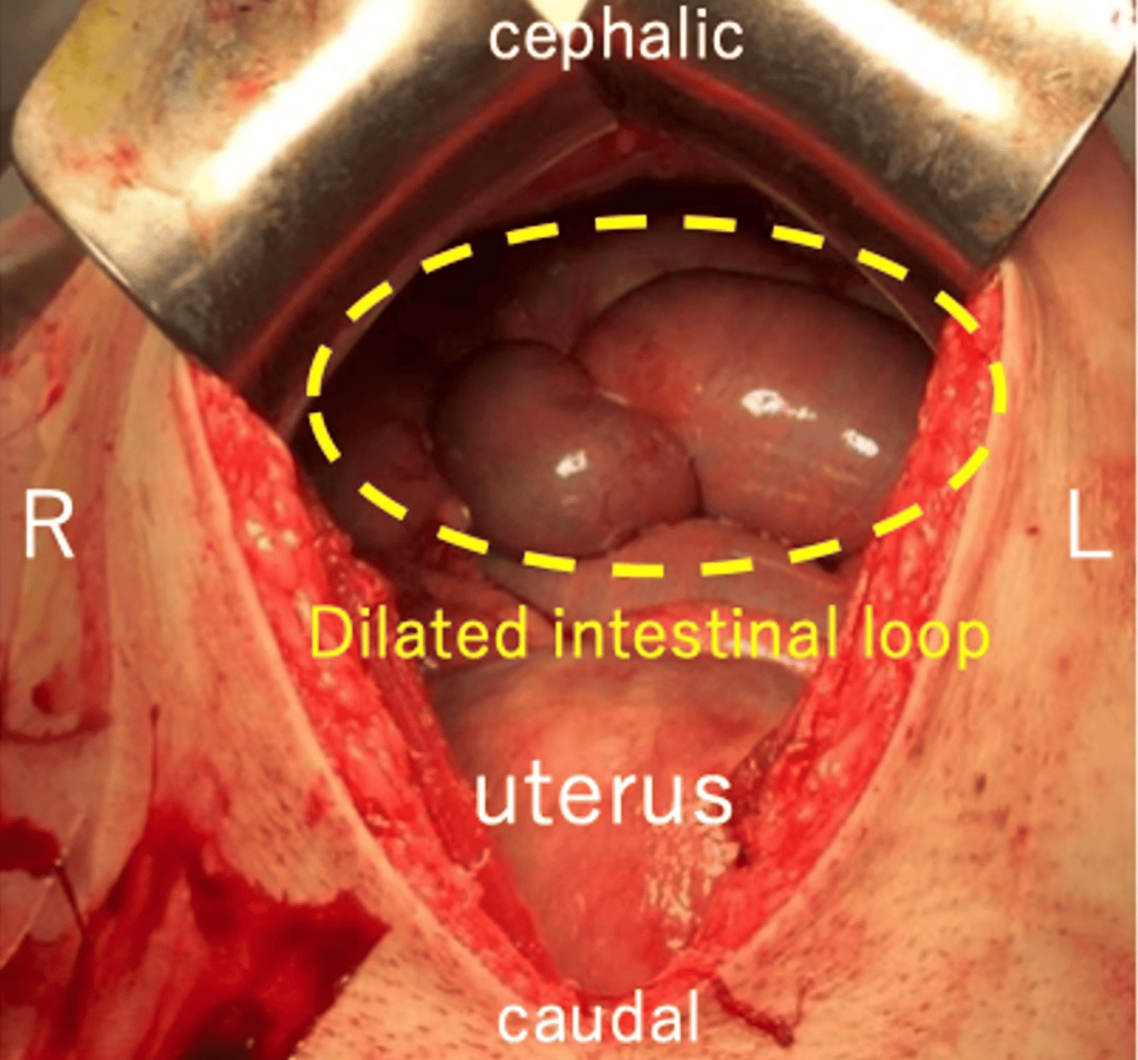
Intra-abdominal findings. An enlarged intestinal loop in the upper abdomen is noted.

**Figure 4:**
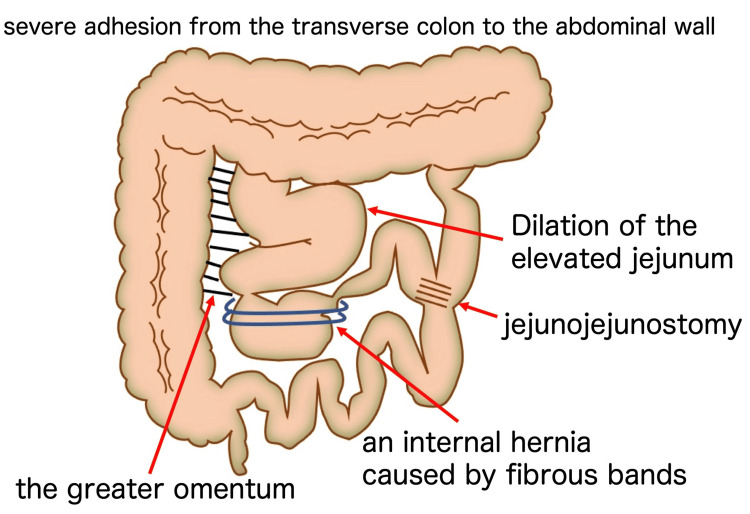
The imaging diagram of the intra-abdominal findings. An internal hernia of the Roux-en-Y limb caused by fibrous bands extending from the greater omentum. The hepaticojejunostomy site is not depicted because of its anatomical location; the jejunum was divided, with the distal limb anastomosed to the hepatic duct and the proximal limb anastomosed to the jejunum (jejunojejunostomy). This illustration was created by the authors and has not been previously published.

After admission, conservative treatment, including fasting, intravenous hydration, and intravenous administration of omeprazole, was initiated. However, the symptoms of epigastric pain and non-bilious vomiting remitted and exacerbated repeatedly. At 35 weeks and six days, the symptoms were further exacerbated without signs of improvement. Hence, induction of labor was initiated at 36 weeks and zero days. An emergency cesarean section with a midline vertical skin incision was performed after induction failure at 36 weeks and one day. The cesarean section was uneventful, and a female baby weighing 2490 g was delivered with Apgar scores of eight and nine at one and five minutes, respectively. Due to the previous history of surgery for CBD, extensive adhesions were present in the upper abdomen, with the transverse colon adhering tightly to the abdominal wall. Exploration of the abdominal cavity was performed, revealing an enlarged intestinal loop in the upper abdomen and an internal hernia of the Roux-en-Y limb caused by fibrous bands extending from the greater omentum (Figure 3). The imaging diagram of the intra-abdominal findings is shown in Figure 4. Although the Roux-en-Y limb showed significant dilation, there was no sign of intestinal necrosis. Therefore, we performed a release of the fibrous bands to relieve the intestinal obstruction. Immediately after surgery, a large amount of bile-stained green stool was observed. Postoperatively, the postoperative course was normal, and the patient was discharged from the hospital on the sixth postoperative day.

## Discussion

To the best of our knowledge, this is the first report of an internal hernia of the Roux-en-Y limb during pregnancy in someone who has a history of Roux-en-Y reconstruction with hepatic jejunal anastomosis for CBD. She showed recurrent epigastric pain and non-bilious vomiting caused by internal herniation and obstructions of the jejunal loop. Intra-abdominal exploration via laparotomy revealed intestinal obstruction attributed to an internal hernia of the Roux-en-Y limb caused by fibrous bands extending from the omentum. Evaluation of intra-abdominal imaging findings (Figure 4) suggested that symptoms of epigastric pain and non-bilious vomiting were likely precipitated by gastrointestinal compression and impaired bile excretion due to intestinal obstruction of the Roux-en-Y limb. Discharging a large amount of biliary green stools immediately after resolution of the Roux-en-Y limb obstruction indicated alleviation of the biliary excretory disturbance. Furthermore, because there was no obstruction of gastrointestinal transit from the stomach to the duodenum, small intestine, and large intestine, normal brown stool was evacuated in spite of the aggravating signs of ileus (recurrent vomiting with epigastric pain).

It was difficult to make a definitive diagnosis of intestinal obstruction in this case, which lacks specific signs and symptoms for intestinal obstruction, such as intestinal niveau, small bowel gas, persistent bilious vomiting, and abdominal pain with peritoneal irritation sign. Abdominal ultrasound is often used in pregnant women because it is considered safe, but difficulties in assessing abdominal findings due to intestinal gas and uterine enlargement likely contributed to diagnostic complexity [6]. In this case, although a definitive diagnosis of bowel obstruction could not be made preoperatively, we performed an emergency cesarean section through a vertical midline incision with the possibility of a bowel obstruction in mind, which resulted in the discovery of the obstruction of the Roux-en-Y limb in the upper abdomen.

Similar cases of intestinal obstruction due to internal hernia during pregnancy in patients who underwent Roux-en-Y gastric bypass (RYGB), a globally popular bariatric surgery for morbid obesity, have been reported previously [4,7]. Internal hernia has been described in the literature as the most common late complication after RYGB, constituting the primary cause of postoperative bowel obstruction, accounting for 42% of the cases [8-10]. As observed in the present case, pregnant women after Roux-en-Y anastomosis surgery are especially susceptible to intestinal obstruction due to internal hernia, caused by increased abdominal pressure and cephalic deviation of the bowel associated with uterine enlargement. Additionally, an internal mesenteric defect due to adhesion between the transverse colon mesentery and small bowel mesentery after Roux-en-Y anastomosis could be the cause of an internal hernia of the small intestine [11].

Intestinal obstruction due to an internal hernia in the Roux-en-Y limb generally lacks typical symptoms suggestive of intestinal obstruction. These cases are prone to being misdiagnosed as reflux esophagitis, gastritis, or pancreatitis, which can lead to rapid progression to the strangulation and ischemia of the Roux-en-Y limb [7]. According to Phillip et al., 98% of pregnant women with bowel obstruction experienced abdominal pain, 82% showed vomiting, and 71% had tenderness on palpation [12]. Overall maternal and fetal mortality rates were 6% and 26%, respectively, indicating the need for early intervention in cases of bowel obstruction during pregnancy [12]. In the present case, the symptoms and signs were nonspecific, and reflux esophagitis was initially suspected. If the intra-abdominal survey at laparotomy had been delayed, it could have developed into necrosis of the intestinal tract, necessitating intestinal resection, which could have had adverse outcomes in the mother-infant dyad.

## Conclusions

In conclusion, if a pregnant woman with a history of Roux-en-Y reconstruction experiences recurrent epigastric pain and non-bilious vomiting but with normal brown stool, the potential progression to Roux-en-Y limb obstruction and small bowel strangulation should be kept in mind. Given the serious adverse consequences for both mother and child due to treatment delays, collaboration between surgeons and obstetricians is crucial for determining the appropriate timing for the surgical intervention for those mothers.
